# Nondomain biopolymers: Flexible molecular strategies to acquire biological functions

**DOI:** 10.1111/gtc.13050

**Published:** 2023-05-30

**Authors:** Kazuharu Arakawa, Tetsuro Hirose, Toshifumi Inada, Takuhiro Ito, Toshie Kai, Masaaki Oyama, Yukihide Tomari, Takao Yoda, Shinichi Nakagawa

**Affiliations:** ^1^ Institute for Advanced Biosciences Keio University Tokyo Japan; ^2^ RNA Biofunction Laboratory, Graduate School of Frontier Biosciences Osaka University Suita Japan; ^3^ Division of RNA and Gene Regulation, Institute of Medical Science The University of Tokyo Tokyo Japan; ^4^ Laboratory for Translation Structural Biology RIKEN Center for Biosystems Dynamics Research Yokohama Japan; ^5^ Germline Biology Laboratory, Graduate School of Frontier Biosciences Osaka University Osaka Japan; ^6^ Medical Proteomics Laboratory, The Institute of Medical Science The University of Tokyo Tokyo Japan; ^7^ Laboratory of RNA Function, Institute for Quantitative Biosciences The University of Tokyo Tokyo Japan; ^8^ Nagahama Institute of Bio‐Science and Technology Nagahama Japan; ^9^ RNA Biology Laboratory, Faculty of Pharmaceutical Sciences Hokkaido University Sapporo Japan

**Keywords:** intrinsically disordered proteins, noncoding RNA, nondomain biopolymers, nonmembranous organelles, nuclear bodies, phase separation

## Abstract

A long‐standing assumption in molecular biology posits that the conservation of protein and nucleic acid sequences emphasizes the functional significance of biomolecules. These conserved sequences fold into distinct secondary and tertiary structures, enable highly specific molecular interactions, and regulate complex yet organized molecular processes within living cells. However, recent evidence suggests that biomolecules can also function through primary sequence regions that lack conservation across species or gene families. These regions typically do not form rigid structures, and their inherent flexibility is critical for their functional roles. This review examines the emerging roles and molecular mechanisms of “nondomain biomolecules,” whose functions are not easily predicted due to the absence of conserved functional domains. We propose the hypothesis that both domain‐ and nondomain‐type molecules work together to enable flexible and efficient molecular processes within the highly crowded intracellular environment.

## Graphical abstract

This review challenges the long‐held belief in molecular biology that the functionality of biomolecules solely hinges on conserved sequences and their resultant structures. Instead, we explore the roles and molecular mechanisms of “nondomain biomolecules,” which lack conserved functional domains and utilize inherent flexibility for their functional roles. Our hypothesis suggests that both domain and nondomain biopolymers cooperate to facilitate flexible and efficient molecular processes in the highly crowded intracellular milieu.

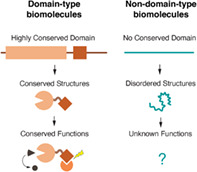

## 1 EMERGING WORLD OF NONDOMAIN BIOPOLYMERS

**FIGURE 1:**
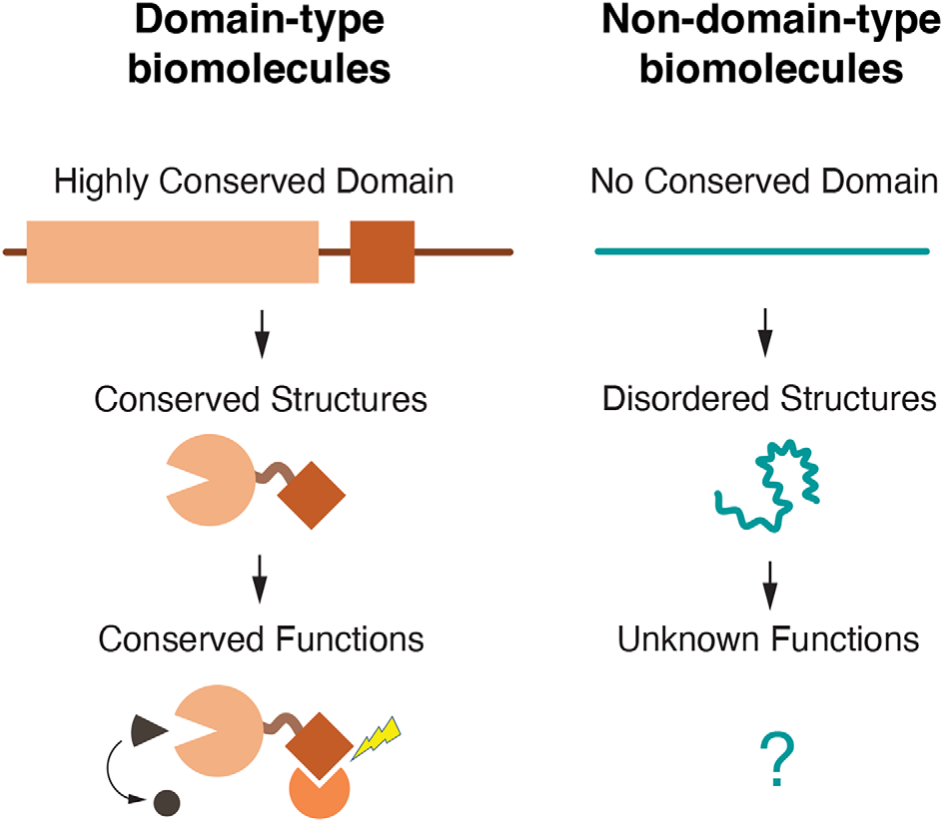
Concept of domain‐type biomolecules and nondomain‐type biomolecules. Highly conserved primary sequences typically fold into conserved structures and perform conserved functions. Under this dogma, the functions of biomolecules or regions with nonconserved sequences have often been underestimated. Nondomain‐type biomolecules represent a unique group of biomolecules whose functions may be less dependent on their primary sequences and defined structures.

Over the past few decades, substantial advancements in molecular biology have established that conserved biomolecules, such as proteins and nucleic acids, fold into homologous tertiary structures and mediate analogous functions (Figure 1). Consequently, even when encountering novel genes, analysis of the domain organization of uncharacterized gene products has enabled systematic functional predictions and rational experimental design to elucidate precise molecular mechanisms. Following Jacques Monod's renowned declaration, “What is true for *Escherichia coli* is also true for elephants,” molecular biology has made remarkable progress by analyzing homologous biomolecules across a wide range of organisms. These “homology studies” have revealed numerous conserved molecular processes and pathways in various fields of cell and molecular biology, including signal transduction pathways that regulate body patterning, molecular pathways that control programmed cell death, and proteasome and autophagy pathways that maintain proteostasis, among others. These studies typically begin with the genetic identification of key molecules in simple model organisms, such as yeast, nematodes, and fruit flies, followed by studies in mammalian species, typically humans and mice. Particularly prevalent during the 1990s and 2000s, these studies have established that the same pathways are utilized in both vertebrates and invertebrates across evolutionarily distant species, with key pathways being repeatedly used in various cellular contexts within the same organisms (Carroll, 2008). In other words, the diversity of molecular pathways underlying complex organisms is simpler than expected, leading to a general consensus that the enumeration of the “molecular parts” that make up a living organism has almost reached a plateau. Indeed, the rate of discovery of novel molecular pathways or their components has slowed over time, causing research trends to shift from identifying new functional genes to quantitatively analyzing global molecular networks to understand biological systems (Kitano, 2002). This trend has been accelerated by the completion of genome sequencing for model multicellular organisms in the 2000s (Adams et al., 2000; C. elegans Sequencing Consortium, 1998; Lander et al., 2001; Mouse Genome Sequencing et al., 2002). Although we now have comprehensive lists of primary protein sequences and transcribed RNAs for these model organisms, an essential question remains: Have we truly obtained a complete catalog of functional biomolecules for living organisms?

Advancements in sequencing technology have led to the discovery of thousands of nonprotein‐coding (noncoding) RNAs transcribed from the genomes of multicellular organisms (Mattick et al., 2023). These noncoding RNAs, larger than 200 nucleotides, are collectively referred to as long noncoding RNAs (lncRNAs). The number of lncRNA‐producing loci is comparable to, or even exceeds, that of protein‐coding mRNA‐producing loci. The lncRNAs are generally expressed in temporally and spatially restricted patterns, primarily localized in the nucleus, and exhibit lower conservation compared with mRNAs (Cabili et al., 2011). Extensive investigations at cellular and individual animal levels have identified diverse functional categories for lncRNAs, including epigenetic regulators of gene expression, architectural components of nonmembranous organelles, molecular sponges, and precursors for small RNAs (Mattick et al., 2023). Despite these efforts, the majority of lncRNAs pervasively transcribed from the genome remain uncharacterized and functionally unvalidated. This can be partially attributed to the absence of common conserved sequence motifs within lncRNAs of the same functional category, in contrast to proteins whose functions can be predicted from conserved sequence domains (Hirose et al., 2014). As a result, our understanding of functional lncRNAs remains incomplete, even in model organisms.

This incompleteness extends to proteins as well, including in simple organisms like yeast. According to a recent analysis (Wood et al., 2019), the number of unknown proteins with unknown functions that cannot be predicted from primary sequences is 676 in fission yeast, 978 in budding yeast, and 3117 in humans, representing 16%, 18%, and 16% of the total number of proteins in each species, respectively. While it is unclear whether these proteins participate in essential biological processes or are merely nonfunctional products of “junk” mRNAs, recent studies have discovered proteins that lack known functional domains but nevertheless have remarkable biological functions, such as tardigrade‐specific proteins that confer extraordinary survival capabilities to these organisms in harsh environments, and heat‐resistant obscure (Hero) proteins that safeguard a diverse array of client proteins (Arakawa, 2022; Tsuboyama et al., 2020). Interestingly, these proteins do not share homologous sequences but they do share a common characteristic: they consist almost entirely of intrinsically disordered regions (IDRs). IDRs, also referred to as low‐complexity regions, contain fewer hydrophobic amino acids that are typically required for the formation of rigid, well‐defined structures. Consequently, IDRs may operate through molecular mechanisms that diverge from those involving structure‐based, specific, and rigid molecular interactions (Oldfield & Dunker, 2014). IDRs have recently attracted significant attention for their ability to mediate multivalent weak molecular interactions and liquid–liquid phase separation (LLPS), providing a molecular basis for the formation of molecular condensates or nonmembranous organelles within cells (Uversky, 2021).

In this review, we refer to proteins and nucleic acids (particularly noncoding RNAs) that have evaded domain‐based functional prediction as “nondomain biopolymers.” We propose that classical structure‐based, highly specific rigid interactions, and multivalent weak interactions mediated by nondomain biopolymers work together within cells to enable efficient and coordinated molecular processes in the highly crowded cellular environment.

## 2 EXAMPLES OF NONDOMAIN BIOPOLYMERS AT WORK

### 2.1 RNA and multivalent weak interactions in nonmembranous organelles

**FIGURE 2:**
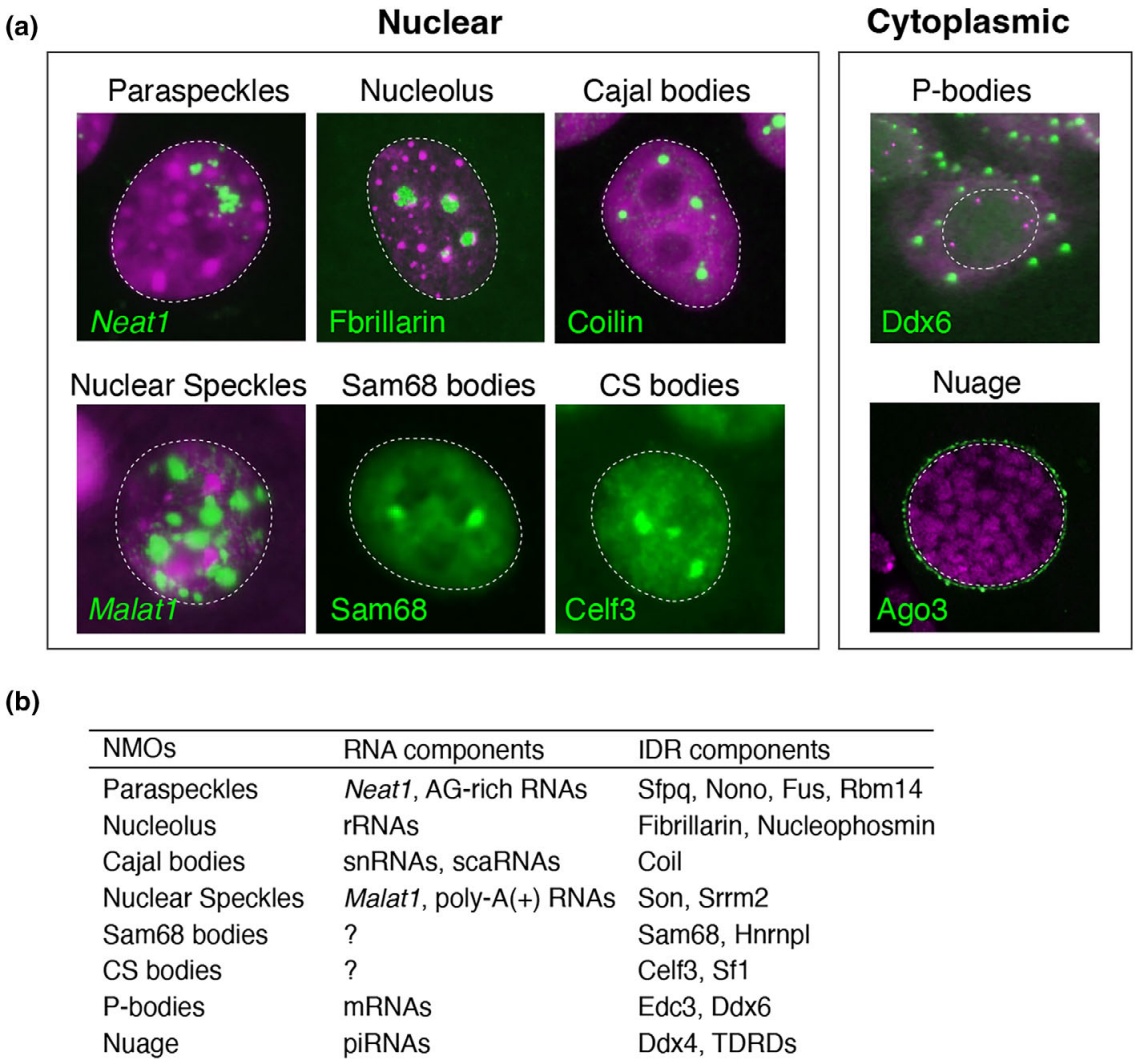
Examples of nonmembranous organelles. (a) Examples of representative nuclear and cytoplasmic nonmembranous organelles. (b) Components of nonmembranous organelles, which typically contain RNA and proteins with distinct intrinsically disordered regions (IDRs).

The cellular nucleus is not a simple container filled with a homogeneous solution of genomic DNA and other nuclear molecules. Instead, each constituent is unevenly distributed, forming distinct submicron‐scale structures called nuclear bodies (Hirose et al., 2023; Pederson, 2011). These structures are also known as nonmembranous organelles or molecular condensates in contemporary terms (Figure 2). Prominent examples include the nucleolus, involved in ribosome biogenesis (Boisvert et al., 2007), Cajal bodies for UsnRNP modification and assembly (Gall, 2000), nuclear speckles enriched with pre‐mRNA splicing regulators (Spector & Lamond, 2011), and paraspeckles regulating cellular differentiation under specific physiological conditions (Nakagawa et al., 2018). Nonmembranous organelles can also be found in the cytoplasm, including P‐bodies and stress granules containing translationally inactive mRNAs and components that regulate translation (Decker & Parker, 2012), as well as nuage or germ granules involved in the processing and biogenesis of piRNAs (Pek et al., 2012). Notably, most nonmembranous organelles contain specific RNA and RNA‐binding proteins with IDRs, suggesting a possible involvement of these molecules in their formation.

**FIGURE 3:**
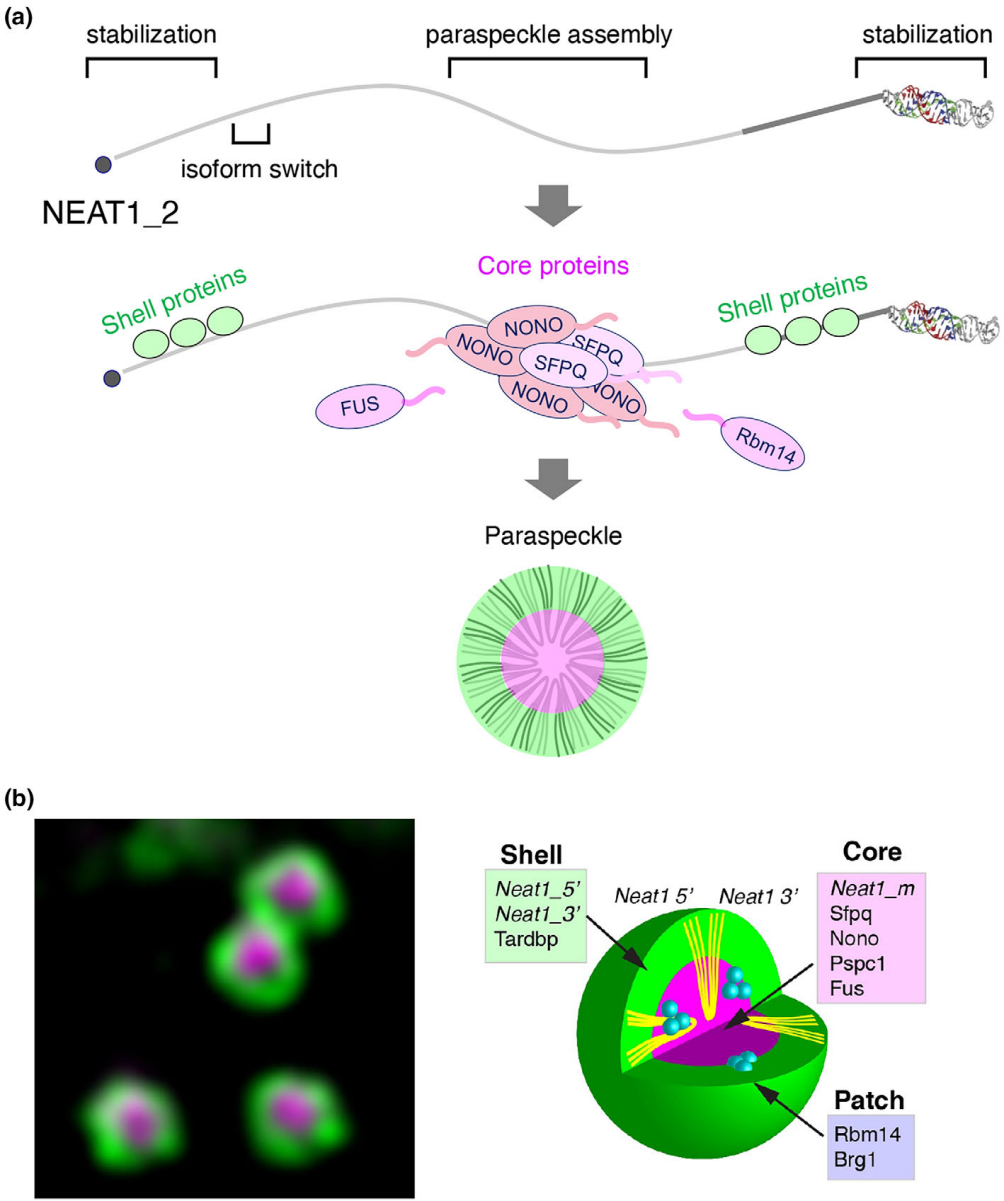
Building of paraspeckles on architectural lncRNA *Neat1*. (a) A model of paraspeckle formation on *NEAT1_2* lncRNA via phase separation. Functional RNA domains are shown by square brackets (top). These domains interact with specific paraspeckle proteins, including NONO and SFPQ, which assemble the core or putative shell‐forming proteins (middle). Multivalent interactions between IDRs of these proteins induce phase separation, leading to the construction of a massive paraspeckle structure (bottom). (b) Core–shell structure of paraspeckles observed under a super‐resolution microscope (left) and schematic drawing of the positions of each component in paraspeckle spheres (right). Images are reprinted from West et al. (2016).

The molecular mechanisms underlying the formation of nonmembranous organelles are best studied in paraspeckles (Figure 3). Paraspeckles were originally described as nuclear bodies enriched in RNA‐binding proteins (RBPs) belonging to the Drosophila Brain and Human Splicing (DBHS) family (Fox et al., 2002). Subsequent research revealed that the long noncoding RNA (lncRNA) *Neat1* (nuclear paraspeckle assembly transcript 1) is exclusively localized to paraspeckles and serves as an architectural component (Chen & Carmichael, 2009; Clemson et al., 2009; Sasaki et al., 2009; Sunwoo et al., 2009). Two isoforms of *Neat1*, *Neat1_1*, and *Neat1_2*, are produced by differential termination of the transcripts, conventional polyadenylation, and RNaseP‐mediated cleavage of tRNA‐like structures, respectively (Naganuma et al., 2012; Wilusz et al., 2012). While both *Neat1* isoforms localize to paraspeckles, only *Neat1_2* can induce their formation (Naganuma et al., 2012; Sasaki et al., 2009). Within paraspeckles, *Neat1_2* transcripts are folded into U‐ or V‐shapes and arranged radially, with 5′ and 3′ ends located peripherally and middle regions located centrally (Souquere et al., 2010; West et al., 2016). A detailed analysis of a series of *Neat1_2* deletion mutants revealed a modular structure for this architectural lncRNA, consisting of the 5′ and 3′ regions required for *Neat1_2* stability, short regions surrounding the polyadenylation site required for *Neat1* isoform switching, and the middle region that induces paraspeckle assembly (Yamazaki et al., 2018). It should be stressed that the paraspeckle assembly region displays low sequence conservation and no consensus sequence motif, making it a typical nondomain biopolymer whose function cannot be easily predicted from primary sequences.

Over 40 proteins are known to be enriched in paraspeckles (Naganuma et al., 2012), binding to specific regions of radially arranged *Neat1_2* and creating core‐shell structures of paraspeckle spheres (West et al., 2016) (Figure 3). Considering that many paraspeckle proteins have distinct IDRs and that IDR‐containing RNA‐binding proteins undergo phase transitions to form gels or liquid droplets under certain conditions in vitro (Hennig et al., 2015; Portz et al., 2021), *Neat1_2* may provide a scaffold for their assembly to increase local concentration, which results in subsequent phase transitions mediated by multivalent weak interactions between IDRs. Indeed, forced recruitment of tandem arrays of Nono replaces the function of the paraspeckle assembly region located at the middle region of *Neat1_2* (Yamazaki et al., 2018). Generally, RNA molecules are inherently flexible and tend to form multiple structures with equal stability in terms of free energy. This property of RNA may be well‐suited for controlling IDR‐mediated multivalent weak interactions, potentially explaining the widespread presence of RNA molecules within nonmembranous organelles (Roden & Gladfelter, 2021). Typically, paraspeckles are submicron‐scale spherical molecular condensates, ~300 nm in diameter. However, their size and shape can vary, and they can fuse to form elongated, sausage‐like structures (Souquere et al., 2010). The dynamic and flexible behavior of paraspeckles can be explained by triblock co‐polymer micelle models (Yamazaki et al., 2021), further supporting the idea that *Neat1* functions as a flexible polymer, rather than a rigid skeleton that strictly assembles associated components.

In summary, the formation of paraspeckles serves as an example of how nondomain biopolymers, specifically lncRNAs and proteins with IDRs, can participate in the assembly and maintenance of nonmembranous organelles. These flexible and dynamic structures rely on multivalent weak molecular interactions and the innate properties of RNA and IDR‐containing proteins to establish and modulate their organization.

### 2.2 Hero proteins

**FIGURE 4:**
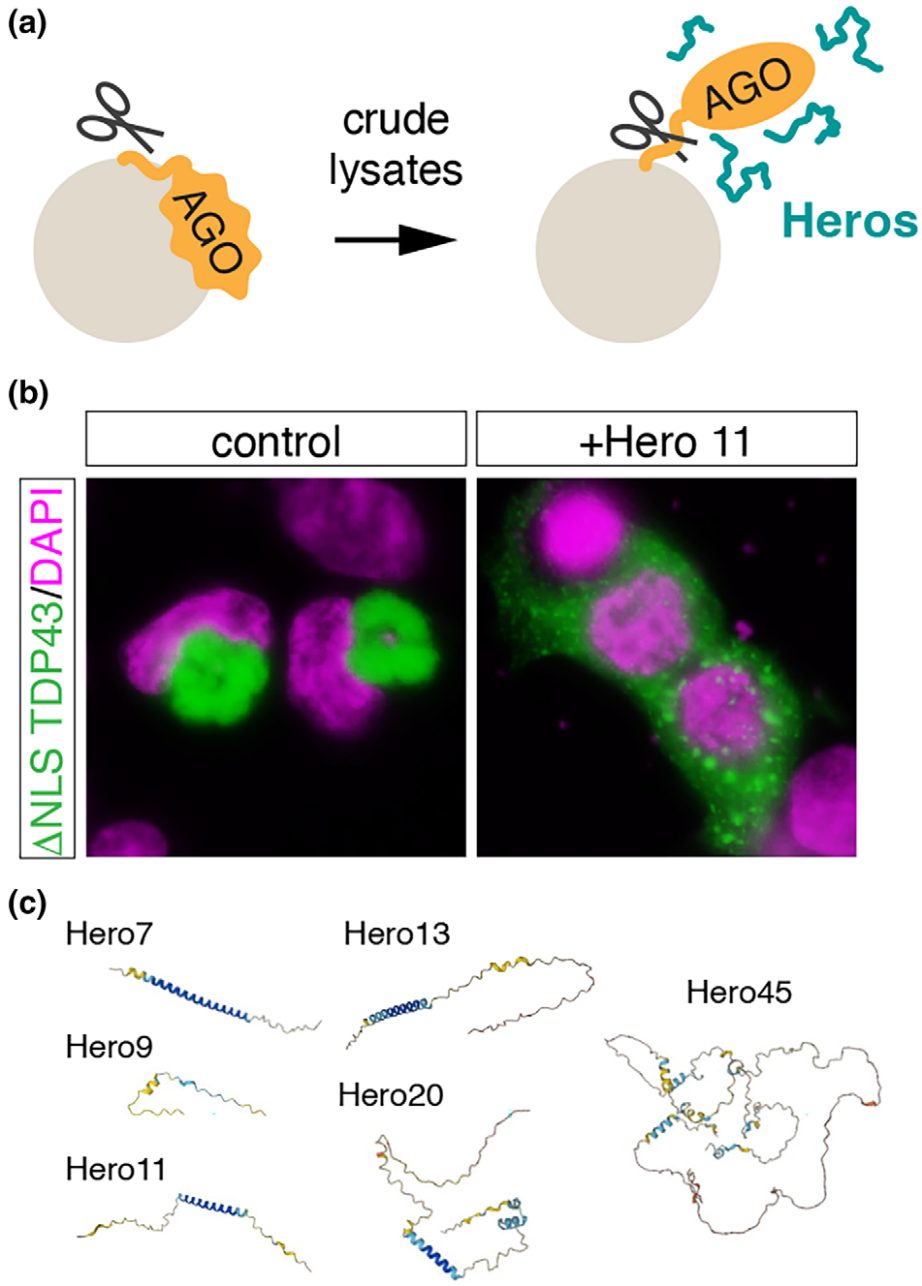
Hero proteins provide an appropriate molecular milieu for various client proteins. (a) Identification of Hero proteins. Hero proteins were originally identified as proteins that assist in the elution of recombinant Ago protein from beads upon protease digestion. (b) Hero11 prevents the formation of cytoplasmic aggregates of TDP‐43 lacking the nuclear localization signal when co‐expressed in cells. (c) Structures of Hero proteins predicted with AlphaFold2. Note that they are highly intrinsically disordered.

The importance of nondomain biopolymers has been further underscored by the serendipitous discovery of a series of heat‐soluble, highly disordered Hero proteins that can protect certain “client” proteins from various stressors (Tsuboyama et al., 2020) (Figure 4). Hero proteins were initially identified as proteins that facilitate the purification of Argonaute (Ago) proteins, an effector protein component of the RNA‐induced silencing complex (Iwakawa & Tomari, 2022). It has been empirically observed that immunopurified Ago protein is difficult to elute from antibody‐conjugated beads. However, the addition of crude cell lysate to the beads considerably increased the recovery of Ago in the soluble fraction (Tsuboyama et al., 2020). This finding suggests that specific factors in the lysate create an environment conducive to Ago proteins adopting an appropriate conformation, resulting in efficient elution from the beads.

The factors that aided in the elution of the immunopurified Ago protein remained soluble and active even after lysate boiling, but their activity was lost upon treatment with proteinase K. This observation indicates that these factors are highly heat‐resistant proteins. Generally, heat resistance is a common characteristic of IDRs that lack a hydrophobic core necessary for the formation of rigid tertiary structures. Subsequent bioinformatic analyses, coupled with mass spectrometry of proteins that remained soluble in boiled cell lysate, led to the identification of a set of poorly characterized proteins that improve the elution of Ago protein from beads. These proteins were named Hero (heat‐resistant obscure) proteins due to their heat‐resistant and disordered properties (Tsuboyama et al., 2020).

Hero proteins not only facilitate the biochemical purification of recombinant Ago proteins, but also protect the activities of a wide range of proteins from various denaturing conditions, including the enzymatic activity of lactate dehydrogenase after dehydration, GFP fluorescence after treatment with an organic solvent, and luciferase activity upon heat shock treatment (Tsuboyama et al., 2020). Moreover, Hero proteins can inhibit the formation of disease‐related aggregates, such as TDP43 and poly GA, when co‐expressed in cells (Tsuboyama et al., 2020; Figure 4b). Unlike protein chaperones that use ATP to refold malformed proteins, Hero proteins do not seem to be able to dissolve pre‐existing protein aggregates. Instead, they act as a buffer or molecular shield that prevents client proteins from forming abnormal conformations in an ATP‐independent manner.

Hundreds of Hero protein candidates, predicted to be intrinsically disordered throughout their entire length, have been identified in both mammals and insects. Currently, six representative human Hero proteins have been demonstrated to possess the remarkable biochemical activities mentioned earlier. These proteins are named based on their predicted molecular weight: Hero7, Hero9, Hero11, Hero13, Hero20, and Hero45 (Tsuboyama et al., 2020). These Hero proteins do not share highly conserved sequence motifs, except for the fact that their intrinsic disorder regions (IDRs) span their entire length. Intriguingly, the ability of Hero7 and Hero11 to inhibit the formation of protein aggregates is preserved even after random shuffling of their primary amino acid sequences (Tsuboyama et al., 2020). This outstanding finding suggests that their function is determined by amino acid composition rather than protein structure, a mechanism that starkly contrasts with conventional proteins that rely on their tertiary structures for functionality.

Although there are no sequence motifs conserved across all Hero proteins, it is important to note that the primary sequence of each Hero protein is somewhat conserved between species. Furthermore, some of their functions have been studied in other organisms and experimental systems. For instance, Hero7 (SERF2) was first identified in *Caenorhabditis elegans* as MOAG‐4, which can enhance the aggregation of exogenously expressed aggregation‐prone proteins (van Ham et al., 2010). Hero45 is a homologue of Stm1 (suppressor of tom 1), discovered as a multicopy suppressor of genes involved in mitosis in budding yeast (Utsugi et al., 1995). Hero45 has also been described as PAI‐RBP1 (plasminogen activator inhibitor mRNA binding protein 1) or Serbp1 (Serpin1 mRNA binding protein 1) (Heaton et al., 2001). This protein binds to ribosomes and obstructs the mRNA entrance channel (Anger et al., 2013; Brown et al., 2018).

### 2.3 Tardigrade‐specific highly disordered proteins

Tardigrades, also known as water bears, are microscopic, eight‐legged animals belonging to the phylum Tardigrada that inhabit wet environments or water. Some tardigrade species, like *Ramazzottius varieornatus* (Horikawa et al., 2008), undergo a rapid physical and morphological transformation upon desiccation to form the “tun state.” This state enables them to survive extreme nonphysiological conditions, including temperatures up to 100°C, ionizing radiation of 3000–5000 Gy which is 1000 times the lethal dose for humans, and exposure to the vacuum of outer space. Once rehydrated, these animals quickly regain their original physiology and resume their previous activities (Arakawa, 2022).

**FIGURE 5:**
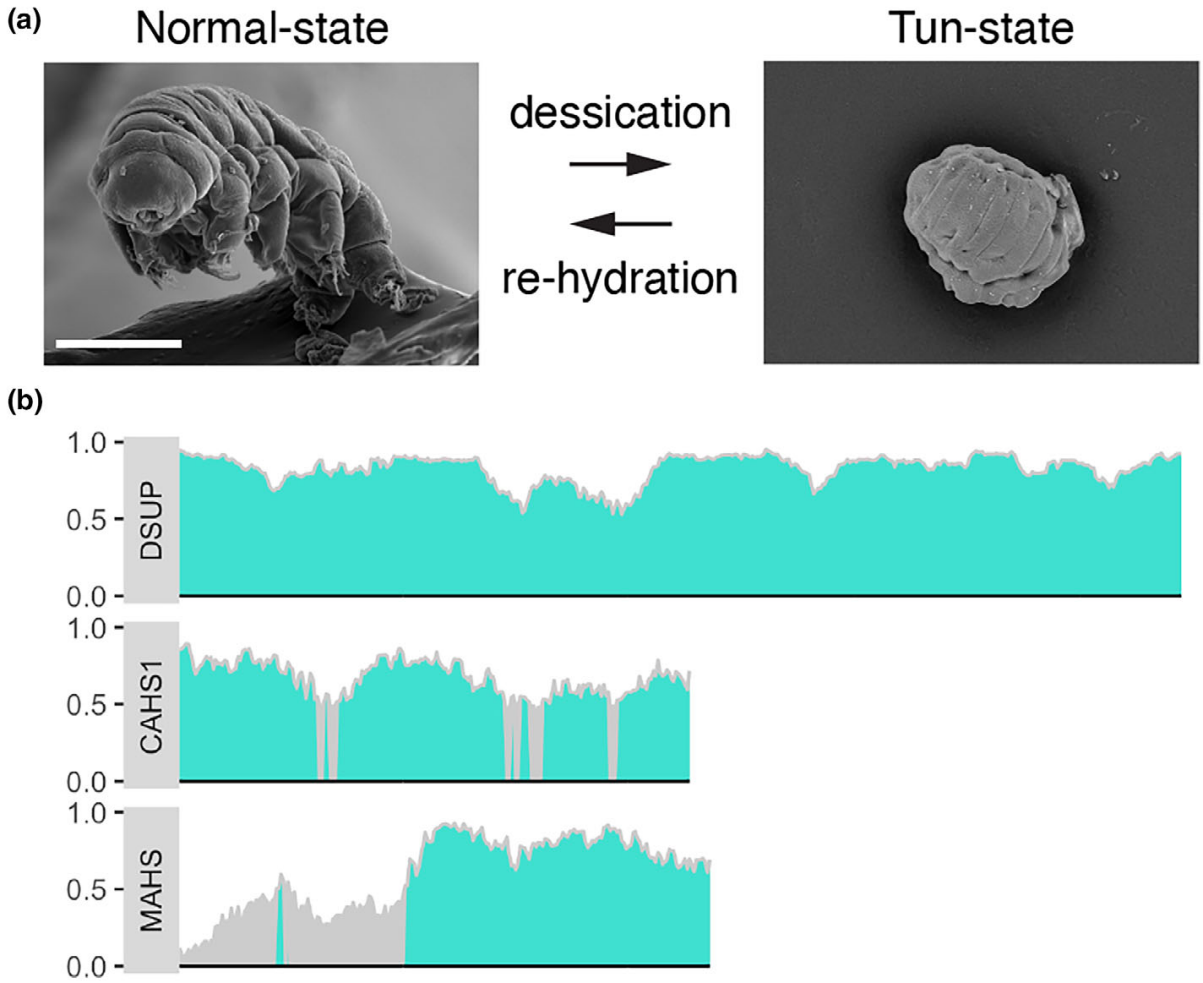
Tardigrades' extreme resistance to harsh environments. (a) Scanning electron microscope images of a tardigrade (water bear) in both the living and tun‐state. Images are reprinted from Arakawa (2022). (b) Prediction of intrinsically disordered regions in tardigrade‐specific proteins associated with the tun‐state.

Unlike other animals, such as brine shrimp, which rely on trehalose to survive desiccation, tardigrades achieve the tun state through a set of highly disordered proteins unique to them (Arakawa, 2022; Figure 5). Studies in plants have identified proteins called late embryogenesis abundant (LEA) proteins that facilitate cell survival under anhydrous conditions. LEA proteins are a group of hydrophilic proteins that are highly expressed during the late stages of seed development and in response to various stresses such as drought, cold, heat, and high salinity. One of the key features of LEA proteins is their heat‐solubility, which led researchers to search for several heat‐soluble, highly disordered, tardigrade‐specific proteins that might be involved in the remarkable ability of this animal to survive harsh environments. This led to the identification of a series of highly disordered proteins that localize to different subcellular compartments, including cytoplasmic abundant heat‐soluble (CAHS; Yamaguchi et al., 2012), mitochondrial abundant heat‐soluble (MAHS; Tanaka et al., 2015), and damage suppressor (Dsup; Hashimoto et al., 2016).

Dsup, a nuclear protein, localizes to the nucleus when exogenously expressed in mammalian cells. Its trans‐species overexpression provides mammalian cells with increased resistance to X‐ray irradiation. Dsup also binds to nucleosomes in vitro, protecting DNA from radical‐mediated cleavage (Chavez et al., 2019). Although putative Dsup genes can be found in several tardigrade species' genomes, their sequence homology is too low for conventional BLAST search algorithms to identify (Yoshida et al., 2017). The only common feature among these Dsup family proteins is their highly disordered structure, similar to Hero proteins, and their locations in the syntenic regions of the genomes when compared between different tardigrade species.

The physiological functions of CAHS and MAHS have yet to be validated. However, several lines of evidence suggest they actively contribute to tardigrades' tun state survival abilities in extreme environments (Arakawa, 2022). First, these proteins are constitutively expressed in tardigrade species that can enter the tun state immediately upon acute desiccation, whereas they are conditionally upregulated in species that require a certain period of time under mildly dehydrated conditions to prepare for the tun state. Second, CAHS are highly abundant, comprising 5%–20% of mRNAs in animals in the tun state. Third, under osmotic stress, CAHS undergoes rapid intermolecular assembly to form a reversible fibrillar structure, which may reflect its protective function during dehydration (Yagi‐Utsumi et al., 2021). Notably, analyses of multiple tardigrade species' genomes revealed hundreds of novel tardigrade‐specific proteins with upregulated expression upon dehydration. Many of these proteins are intrinsically disordered. Tardigrades may be considered a group of animals that maximize the use of nondomain biopolymers to survive in harsh environments. Recently, it has become feasible to introduce exogenous genes in tardigrades, which should enable functional analyses of these tardigrade‐specific genes in the future (Tanaka et al., 2023).

In summary, tardigrades present a unique model for investigating the role of nondomain biopolymers, specifically intrinsically disordered proteins, in adapting to extreme environments. The study of these proteins, such as CAHS, MAHS, and Dsup, offers valuable insights into the mechanisms underlying their protective functions and could potentially lead to the development of novel biotechnological applications. As our understanding of tardigrade biology advances, and with the ability to introduce exogenous genes into these organisms, researchers will have the opportunity to further investigate the roles and functions of these proteins and other tardigrade‐specific genes. This knowledge could provide innovative approaches for enhancing the resilience and survival of other organisms, for the development of dry preservation technologies for biological materials, and for improving our understanding of the biological strategies employed by extremophiles to endure harsh environments.

## 3 MOLECULAR MECHANISMS OF NONDOMAIN BIOPOLYMERS

Nondomain biopolymers refer to a group of biomolecules, including lncRNAs and IDPs, whose functions and structures are difficult to predict based on known functional domain organization. Although they do not share a common molecular mechanism, they can be categorized into at least three groups according to their mode of action.

### 3.1 Nondomain biopolymers as regulators of phase transitions

**FIGURE 6:**
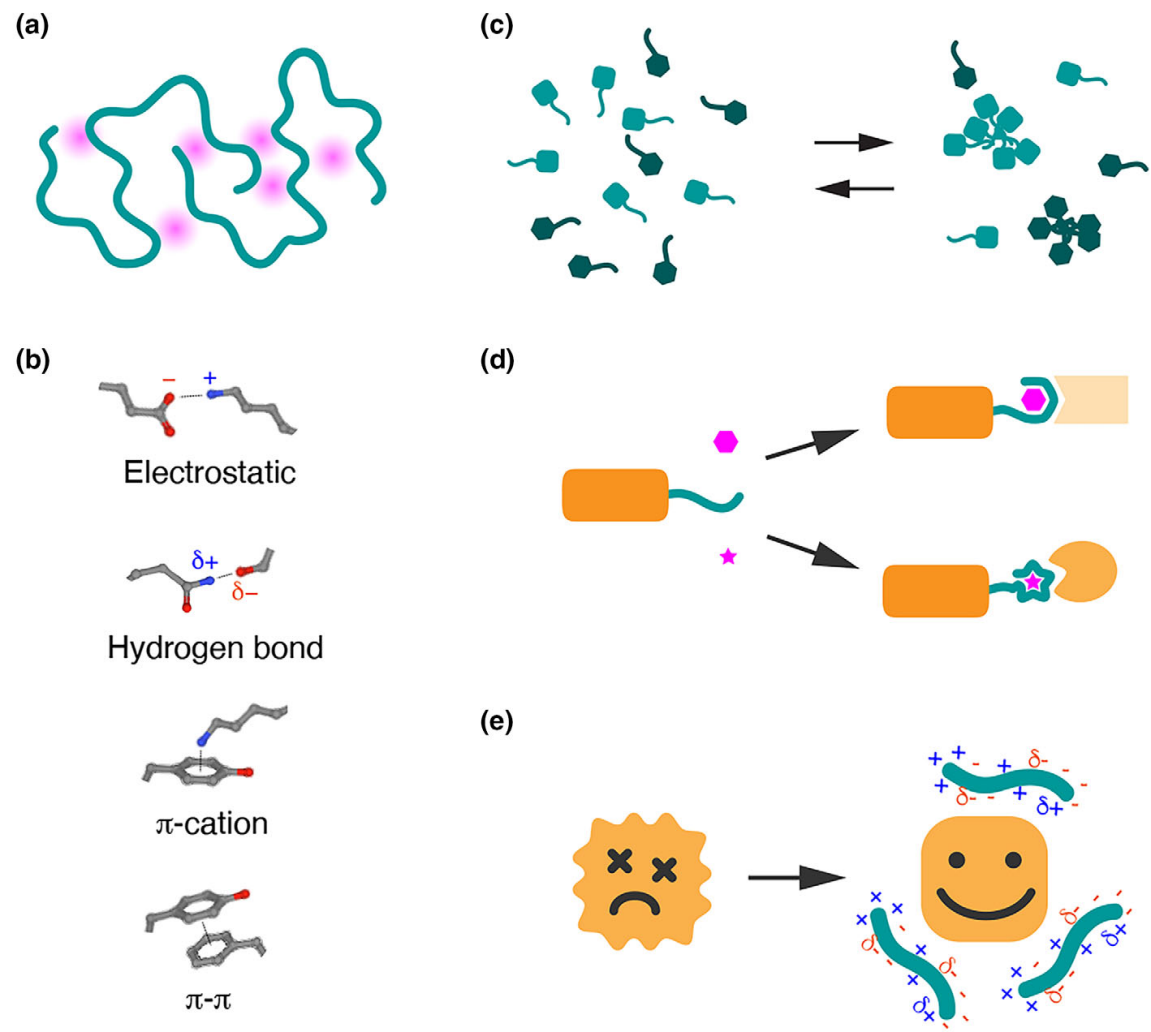
Molecular mechanisms of nondomain biopolymers. (a) A schematic model illustrating the multivalent interactions of nondomain biopolymers. (b) Typical molecular interactions involved in multivalent interactions. (c) Formation of molecular condensates by nondomain biopolymers. (d) Formation of specific structures by nondomain biopolymers upon binding to specific ligands. (e) Nondomain biopolymers function as hydrotropes or molecular shields for client proteins.

First, nondomain biopolymers exert their functions by forming large molecular condensates through multivalent weak interactions (Figure 6a–c). This type of molecular interaction has recently attracted significant attention due to the discovery of nonmembranous organelles like P‐granules behaving like liquid droplets (Brangwynne et al., 2009) and the fact that components of molecular condensates can undergo phase transitions to form liquid droplets or hydrogels under specific in vitro conditions (Feric et al., 2016; Kato et al., 2012). Extensive reviews are available on the importance of multivalent molecular interactions in various cellular processes, including transcriptional regulation, cellular signaling transduction, and protein degradation mediated by proteasomes and autophagy (Boeynaems et al., 2018; Hyman et al., 2014; Kato et al., 2022). Intrinsically disordered regions (IDRs) often contain a high proportion of noncharged polar amino acids, such as glutamine (Q), asparagine (N), serine (S), and tyrosine (Y), which mediate electrostatic interactions and promote interactions with other IDR‐containing proteins. The presence of aromatic residues, like phenylalanine (F), tryptophan (W), and tyrosine (Y), can contribute to π–π stacking and π–cation interactions that stabilize condensates. The specificity of multivalent IDR interactions during molecular condensate formation is not yet fully understood; however, several studies have revealed a molecular “grammar” governing the properties of molecular condensates (Wang et al., 2018). Typically, strong interactions lead to stable complex formations and form hydrogels or more solid aggregates, while transient weak interactions mediate flexible complex formations, resulting in the formation of structures resembling liquid droplets. Furthermore, valency and frequency of aromatic residues regulate phase behaviors of IDRs (Martin et al., 2020). The distribution of positively and negatively charged amino acids within IDRs can also influence interaction specificity, as recently demonstrated for the transcription activator domain of transcription factors (Lyons et al., 2023). It should be noted that classical highly specific interactions between structured domains can also contribute to molecular condensate formation. For example, SH3 domains recognize PxxP motifs in associating proteins, and induce phase separation when multiple domains are connected with flexible linker regions (Harmon et al., 2017). The presence of “stickers” that confer multivalent interactions and flexible “linkers” connecting multiple stickers may be hallmarks for IDRs undergoing phase transitions, which can be explained using coarse‐grained molecular dynamic simulations (Harmon et al., 2017). RNA molecules also contribute to IDR‐mediated molecular condensate formation by providing a scaffold for IDR‐containing RNA‐binding proteins, enabling multivalent interactions that would not normally occur at cellular concentrations (Roden & Gladfelter, 2021). Furthermore, RNA molecules themselves have an intrinsic property to form molecular condensates due to their divalent nature, with both sticker and linker characteristics. RNA can form multiple secondary structures, functioning as a flexible linker, while simultaneously binding to specific targets via complementary sequences, functioning as stickers.

### 3.2 Nondomain biopolymers involved in coupled folding

Second, nondomain biopolymers can form stable molecular complexes by binding to specific ligands or proteins, creating functional structures (Figure 6d). This “coupled folding” concept has long been recognized in the transactivating domains of transcription factors, which often consist of intrinsically disordered regions without homology to other transcription factors or family proteins (Minezaki et al., 2006). These unstructured regions can adopt stable conformations upon binding to specific ligands or partner proteins, allowing versatility and adaptability in protein–protein interactions. This attribute facilitates recruitment and assembly of transcriptional machinery, resulting in efficient and precise gene expression control. For example, the tumor suppressor protein p53 has an intrinsically disordered N‐terminal transactivating domain essential for its function. This disordered domain enables p53 to interact with various binding partners, including MDM2, RPA, and CBP/p300 (Raj & Attardi, 2017). In the case of cAMP response element‐binding protein (CREB), its transactivating domain, known as the kinase‐inducible domain (KID), undergoes a disorder‐to‐order transition upon binding to the KIX domain of the coactivator CBP/p300 (Sugase et al., 2007). Coupled folding can also be observed in the regulation of subcellular distribution of chromatin modifiers. DPPA3/Stella/Pgc7, an intrinsically disordered protein without a recognizable functional domain, forms a tight complex with UHRF1, which regulates the maintenance of DNA methylation along with Dnmt1 (Li et al., 2018). DPPA3 undergoes a “disorder‐to‐order” structural transformation and tightly associates with the PHD domain of UHRF1 (Hata et al., 2022). This interaction results in the dissociation of UHRF1 from histones and subsequent nuclear export of this chromatin modifier.

### 3.3 Nondomain biopolymers as hydrotropes or molecular shields

Third, nondomain biopolymers can function as buffers or molecular shields that enhance the solubility of client proteins in various environments (Figure 6e). Considering intrinsically disordered regions (IDRs) are rich in polar and charged amino acids, they may interact with water molecules while also providing hydrophobic patches that associate with other hydrophobic regions in proteins. This dual nature of IDRs can maintain the solubility of aggregation‐prone proteins by shielding surface hydrophobic residues in solution and preventing insoluble aggregate formation. Alternatively, the polar and charged amino acids of IDRs can neutralize the surface charge of client proteins, protecting them from forming large aggregates connected by complementary charges (Tan et al., 2023). These interactions may explain the activity of Hero proteins in inhibiting the formation of disease‐related protein aggregates commonly found in neurodegenerative diseases (Tsuboyama et al., 2020). Notably, Hero7 can inhibit the formation of insoluble aggregates of aggregation‐prone proteins both in vitro and in cultured cells (Tsuboyama et al., 2020), while its *C. elegans* homologue MOAG4 has been identified as a factor promoting polyglutamine aggregation (van Ham et al., 2010). IDRs may act as nucleation centers when inducing protein aggregate formation, while they can also prevent aggregation when functioning as steric shielding. Interestingly, synthetic random copolymers, containing both polar and nonpolar groups, have been shown to coat various protein surfaces and act as surfactants, maintaining solubility and functionality of associating proteins even in non‐natural environments (Panganiban et al., 2018). This function resembles the ability of IDRs to solubilize protein aggregates. In fact, adding intrinsically disordered regions to aggregation‐prone proteins has been shown to increase solubility of fusion proteins (Morimoto et al., 2022; Santner et al., 2012), which can be utilized to help purification of difficult‐to‐express proteins.

In addition to IDRs, RNA can also act as a molecular hydrotrope. Introducing RNase into cells leads to the assembly of aggregation‐prone proteins (Aarum et al., 2020; Maharana et al., 2018). This hydrotrope‐like function of RNA is thought to play a role in maintaining protein solubility within the cell, preventing protein aggregation, and contributing to the formation and dynamics of biomolecular condensates.

## 4 CONCLUSIONS AND FUTURE PERSPECTIVES

As previously mentioned, even 20 years after the completion of the human and mouse genomes, hundreds of protein‐coding genes remain functionally unannotated due to a lack of conserved sequences homologous to known proteins (Wood et al., 2019). Moreover, the functions of tens of thousands of long noncoding RNAs (lncRNAs) pervasively transcribed from the genome are largely unknown (Mattick et al., 2023). Interestingly, recent ribosome profiling analyses have revealed that significant portions of these lncRNAs are associated with translating ribosomes, suggesting that they produce novel peptides or small proteins that escaped computational annotations (Ingolia et al., 2019). Ribosome profiling analyses have also uncovered nonclassical translation products from 5′ or 3′ untranslated regions of known mRNAs, referred to as uORFs and dORFs, respectively (Lee et al., 2021; Wu et al., 2020). Indeed, transcriptome‐based mass spectrometry analyses (Frith et al., 2006; Oyama et al., 2004) and individual experimental detections of each atypical ORF product using tag‐insertion have confirmed the existence of such peptides, most of which possess novel sequences that exhibit no homology to known protein families. Mutational analyses have revealed that these novel peptides actually play crucial physiological roles in animals (Matsumoto & Nakayama, 2018). Highly sensitive proteomic methodologies based on post‐translational modification (PTM)‐directed biochemical enrichment have also enabled us to detect and quantify site‐specific PTM dynamics of each novel molecule (Kozuka‐Hata et al., 2012). Along with bona fide lncRNAs that are not translated into proteins, there remains a large number of nondomain biopolymers produced from the genome, which potentially exert their functions via mechanisms that have not yet been clarified. It is essential to conduct genome‐wide screenings, especially focusing on these unannotated products, using CRISPRi or other techniques, in order to fully understand the potential of the genomes and the molecular processes regulated by their output products. Classical in‐depth analyses of individual gene products using mutant animals also provide invaluable information on the physiological significance of unannotated nondomain biopolymers.

Considering that nondomain biopolymers may exert their functions via molecular mechanisms distinct from conventional rigid biopolymers that interact with other biopolymers in a highly specific manner, we might have to employ new techniques to investigate their molecular mechanisms. Because multivalent weak interactions may be lost during conventional biochemical purification of tightly associated complexes (e.g., washing steps during immunoprecipitation), proximity labeling methods such as Bio‐ID or cross‐linking immunoprecipitation will be required to investigate client or partner proteins of nondomain biopolymers. Considering that nondomain biopolymers typically do not form rigid structures and cannot be analyzed by conventional crystallography approaches, molecular dynamics (MD) simulations may also become indispensable tools in understanding the intricate behavior of these biomolecules, as has been shown for the analysis of the interaction between Hero11 and TDP43 (Tan et al., 2023).

By definition, the functions of nondomain biopolymers cannot be predicted from their primary sequences. Currently, we can roughly predict the tendency not to form defined structures using various IDR predictors, including IUPred2A (Meszaros et al., 2018), SPOT‐Disorder2 (Hanson et al., 2019), PONDR (Obradovic et al., 2003), and AlphaFold2 (Jumper et al., 2021), which are useful as initial screening tools for potential candidates of functional nondomain proteins. Classical secondary structure predictions for RNA also provide some clues for predicting nondomain RNAs. Recently, deep mutagenesis analyses have become feasible, allowing for the testing of every possible mutation for each residue (Fowler & Fields, 2014). Future bioinformatic analyses, combined with a deep learning approach, may enable predictions of precise rules governing the composition and sequence of nondomain biopolymers.

In conclusion, the structural flexibility and adaptability of nondomain biopolymers may offer a unique advantage in the process of evolution. These biomolecules, with their ability to undergo coupled folding and form versatile interactions, might have enabled rapid adaptation to changing environments or facilitated the acquisition of novel functions. The lack of conserved sequences in nondomain biopolymers could allow for greater sequence variability and tolerance for mutations, leading to the emergence of new functional variants. This is particularly relevant when considering the large number of unannotated lncRNAs and novel peptides or small proteins produced from nonclassical translation products, which may contribute to the evolution of new cellular functions. Studying nondomain biopolymers, therefore, may provide insights into the mechanisms by which organisms evolve and adapt to diverse environments and challenges. As our understanding of these unique biomolecules improves, it will be crucial to explore their potential roles in evolution and their contributions to the functional diversity of cellular systems.
